# Case Report: Reversible alien hand syndrome caused by cerebral infarction

**DOI:** 10.3389/fnhum.2025.1551539

**Published:** 2025-03-12

**Authors:** Guo-Liang Lin, Xiao-Qian Yu, Han-Yu Cai, Ru-Yi Zhou, Xiao-Tian Li, Xiong Zhang, Jian-Yong Wang

**Affiliations:** Department of Neurology, Institute of Geriatric Neurology, The Second Affiliated Hospital and Yuying Children’s Hospital, Wenzhou Medical University, Wenzhou, China

**Keywords:** alien hand syndrome, corpus callosum, stroke, involuntary movements, apraxia

## Abstract

**Introduction:**

Alien hand syndrome (AHS) is a rare apraxia syndrome that may arise from several neurological disorders including stroke. Given the uncommon symptoms, stroke with AHS as its main manifestation often results in diagnostic challenges and treatment delays.

**Case presentation:**

We herein presented a case of post-stroke AHS caused by corpus callosum infarction. We prescribed him aspirin, clopidogrel, atorvastatin and memantine, and his AHS was remitted completely within 8 days.

**Conclusion:**

AHS is a rare manifestation of cerebral infarction that is generally reversible. Rapid identification of post-stroke AHS and early initiation of treatment are important to improve patient’s prognosis.

## Introduction

Stroke is a common neurological disease characterized by the acute onset of neurological deficits (Hilkens et al., 2024). Rapid diagnosis and reperfusion therapy of ischemic stroke are very important for reducing disability. However, some uncommon symptoms make stroke diagnosis difficult, such as post-stroke movement disorders (Suri et al., 2018; Gupta and Pandey, 2018), especially when they are the main symptoms of stroke.

Alien hand syndrome (AHS) is a rare and confusing neurological disorder characterized by involuntary movements of the affected limb as if it does not belong to the patient, that is often accompanied by intermanual conflict (Manea et al., 2024; Hassan and Josephs, 2016). AHS may arise from corticobasal syndrome, hereditary diffuse leukoencephalopathy with axonal spheroids, Lewy body dementia and Creutzfeldt-Jakob disease (Graff-Radford et al., 2013). Cerebrovascular disease is also a rare cause of AHS (Ma et al., 2023).

Herein, we report a case of a male patient with acute onset of AHS, which was caused by ischemic stroke.

## Case presentation

**Figure 1:**
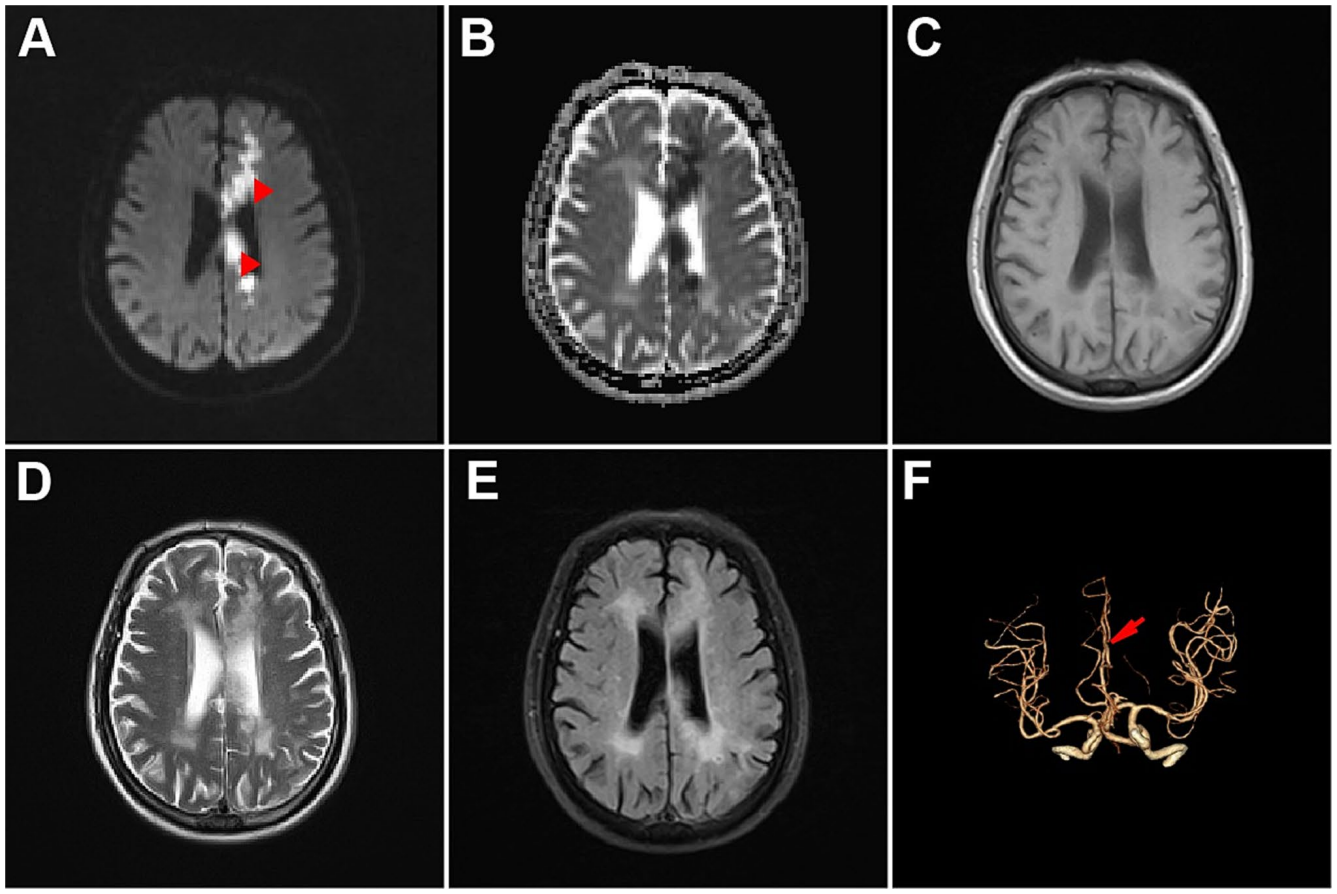
Brain imaging of the patient. **(A)** Diffusion-weighted image. **(B)** Apparent diffusion coefficient image. **(C)** T1-weighted image. **(D)** T2-weighted image. **(E)** Fluid attenuated inversion recovery image. **(F)** Brain computed tomography angiography image.

**Figure 2:**
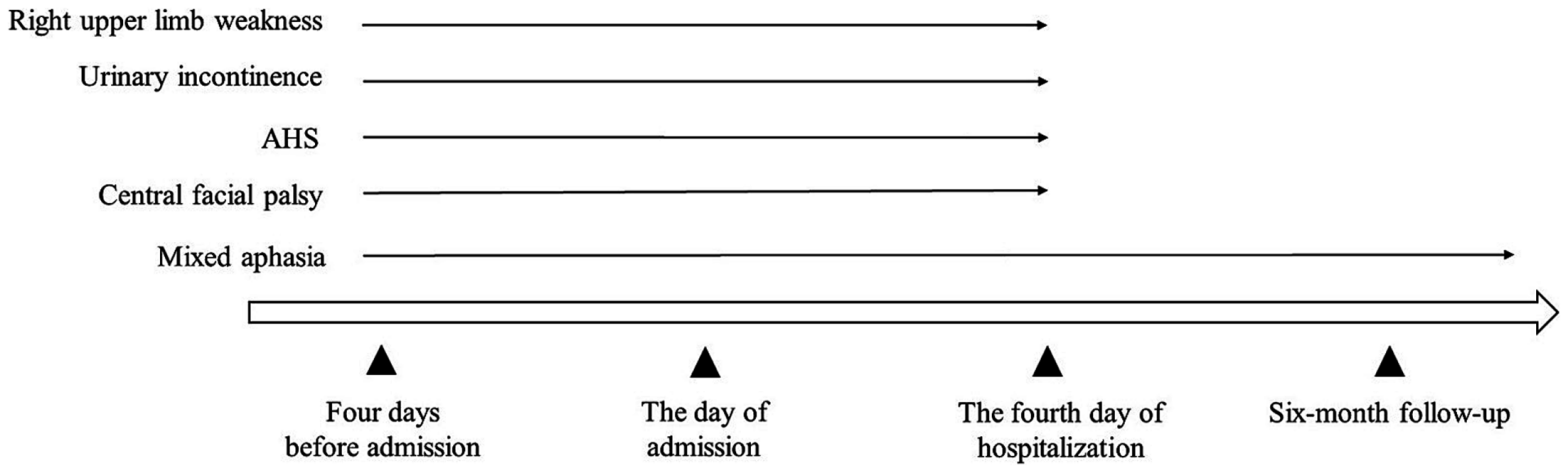
Timeline of the patient’s symptoms. AHS, Alien hand syndrome.

The patient was a 71-year-old right-handed Chinese man with a medical history of hypertension and diabetes mellitus. He had no neurological disorders such as dementia or aphasia. The patient was transported to the emergency department of the Second Affiliated Hospital of Wenzhou Medical University because he began to experience sudden-onset urinary incontinence, aphasia and involuntary movements of his right upper limb 4 days ago. During the 4 days, the patient’s family did not send the patient to the hospital for treatment because they did not realize the seriousness until they found that the patient was completely unable to speak and could not understand what others were saying. Neurological examinations revealed that the patient was alert but in mixed aphasia. Central facial palsy was found on his right face. The muscle strength of his right upper limb was mildly reduced, and the muscle strength of the remaining limbs was normal (Table 1). The sensory function and deep tendon reflex of the patient were normal. The Babinski sign was present on the right side. Continuous involuntary movements were found in his right hand which seemed against his will, while his left hand attempted to stop his right hand (Supplementary Video 1). Other neurological signs were unremarkable. Brain magnetic resonance imaging revealed acute cerebral infarction affecting the left frontoparietal lobe, cingulate gyrus and corpus callosum (Figures 1A–E). Computed tomography angiography showed occlusion of the left anterior cerebral artery (Figure 1F), which was consistent with the area of cerebral infarction. Electrocardiogram and blood tests including complete blood count and coagulation indices were within normal range. We diagnosed the patient with acute cerebral infarction manifesting AHS, which was caused by occlusion of the left anterior cerebral artery. Considering that his cerebral infarction was caused by major intracranial artery occlusion, we prescribed him antiplatelet drugs aspirin and clopidogrel, as well as atorvastatin for lowering low-density lipoprotein cholesterol (Kleindorfer et al., 2021). We also prescribed him memantine to improve post-stroke aphasia (Zhang et al., 2018). His clinical conditions were gradually improved, and the AHS was remitted completely on the fourth day of hospitalization. At 6-month follow-up after discharge, the patient remained aphasic but no longer suffered from AHS (Figure 2). The patient and his family are satisfied with the treatment.

**Table 1:** Muscles evaluations of the patient.

Muscle		Score
Shoulder abductors	Left	5
	Right	4
Elbow flexors	Left	5
	Right	4
Wrist extensors	Left	5
	Right	4
Hip flexors	Left	5
	Right	5
Knee extensors	Left	5
	Right	5
Foot dorsiflexors	Left	5
	Right	5
Total		57

## Discussion

AHS is a rare apraxia syndrome that may occur in several different neurological disorders. In cerebrovascular events, unlike typical neurological deficits, AHS mainly manifests as uncontrollable involuntary movements of the affected limbs, that often confuses the diagnosis of stroke and even delays treatment. In the current report, we present a case of a male patient whose AHS was caused by ischemic stroke. His AHS was remitted within 8 days.

AHS has been classified into anterior, callosal and posterior subtypes. They are different in lesion location and clinical manifestations. The anterior subtype is the most common subtype and is mainly caused by lesions of the genu of the corpus callosum or the supplementary motor area (SMA). It most commonly manifests as reflexive grasping and compulsive manipulation of the right upper extremity. Callosal subtype is relatively rare due to the rich vascular supply of the corpus callosum. It is mainly caused by lesions in the corpus callosum or bilateral frontal lobes, and it is characterized by uncoordinated movements of the hands. While the posterior subtype is mainly caused by lesions of non-dominant parietal lobe, thalamus, or medial temporal lobe. Its clinical manifestations mainly include sensation of alien hand and aimless movements (Manea et al., 2024; Hassan and Josephs, 2016).

The mechanism underlying AHS remains unclear. Multiple brain regions including anterior prefrontal cortex, SMA, posterior parietal cortex, anterior cingulate gyrus, thalamus and corpus callosum, as well as connections between brain regions, are believed to be involved in the development of AHS (Manea et al., 2024; Hassan and Josephs, 2016; Sarva et al., 2014). Several different hypotheses have been proposed to explain the phenomenon of AHS. Impairment of SMA, which plays an important role in decision-making, planning, and inhibitory control of actions, may result in compulsive tactile explorations of limbs (Jurgens, 1984). The volitional movement and motor control theory believes that pre-SMA region is related to volitional movements, and its damage will lead to actions without motor planning (Assal et al., 2007). While the interhemispheric disconnection theory holds that lesions in the corpus callosum lead to impairment of bilateral coordinated movement (Berlucchi, 2012). In our case, we believe that the patient’s uncontrolled involuntary movements of the right upper limb and the intermanual conflict of the hands resulted from the interhemispheric disconnection caused by infarction of the left corpus callosum.

It is worth noting that this patient’s AHS relieved within 8 days, while his aphasia persisted. Consistent with ours, a previous study reported 5 cases of post-infarction AHS, whose AHS symptoms resolved within 4–12 days. The study also systematically reviewed all reported AHS cases caused by cerebral infarction involving the corpus callosum and found that their AHS were reversible (Ma et al., 2023). AHS may occur in a series of diseases, including corticobasal syndrome, hereditary diffuse leukoencephalopathy with axonal spheroids, Lewy body dementia, tumor, progressive multifocal leukoencephalopathy, Creutzfeldt-Jakob disease and stroke (Graff-Radford et al., 2013; Hassan and Josephs, 2016; Bahji, 2022). However, it appears to be reversible only after stroke. We speculate that this may be due to the destruction of AHS-related neural networks caused by cerebral infarction (Wolpe et al., 2020), which may be remodeled through subsequent compensation. Therefore, we believe that early reperfusion therapy is of great value to the recovery of AHS.

Treatment of AHS includes sensory tricks, distracting tasks, cognitive behavioral therapy, verbal cues, botulinum toxin A, clonazepam, visualization strategies, and spatial recognition tasks (Sarva et al., 2014). These managements are primarily based on anecdotal reports. The prognosis of post-stroke AHS is the best (Hassan and Josephs, 2016). In this patient, in addition to aspirin, clopidogrel and atorvastatin for ischemic stroke, we also prescribed memantine. It is unclear whether this is effective in alleviating AHS. The treatment of AHS requires more clinical practice and exploration.

In summary, we reported a rare case of reversible AHS caused by cerebral infarction. AHS is different from the common neurological deficits in stroke. So rapid identification and early initiation of treatment are very important to improve patient’s prognosis.

## Data Availability

The original contributions presented in the study are included in the article/Supplementary material, further inquiries can be directed to the corresponding authors.
