# Supplementary material for: Case Report: Reversible alien hand syndrome caused by cerebral infarction
Source: Front Hum Neurosci. 2025 Mar 12;19:1551539. doi: 10.3389/fnhum.2025.1551539 (PMC11936989; doi:10.3389/fnhum.2025.1551539)
Supplement: Supplementary file 1 [file Table_1.docx]

**Supplementary video legend**

**Supplementary video 1.** The patient displayed involuntary movements in his right upper limb and his left hand attempted to stop his right hand.
